# Validation of the 2022 European LeukemiaNet risk stratification for acute myeloid leukemia

**DOI:** 10.1038/s41598-024-57295-5

**Published:** 2024-04-12

**Authors:** Ga-Young Song, Hyeon-Jong Kim, TaeHyung Kim, Seo-Yeon Ahn, Sung-Hoon Jung, Mihee Kim, Deok-Hwan Yang, Je-Jung Lee, Mi Yeon Kim, June-Won Cheong, Chul Won Jung, Jun Ho Jang, Hee- Je Kim, Joon Ho Moon, Sang Kyun Sohn, Jong-Ho Won, Seong Kyu Park, Sung-Hyun Kim, Chang Kyun Choi, Hyeoung-Joon Kim, Jae-Sook Ahn, Dennis Dong Hwan Kim

**Affiliations:** 1 Department of Hematology-Oncology, Chonnam National University Hwasun Hospital, Chonnam National University Medical School, Gwangju, Jeollanam-Do Republic of Korea; 2 Department of Medical Oncology and Hematology, Princess Margaret Cancer Centre, University of Toronto, Toronto, Canada; 3 Department of Computer Science, University of Toronto, Toronto, ON Canada; 4 The Donnelly Centre for Cellular and Biomolecular Research, University of Toronto, Toronto, ON Canada; 5 Genomic Research Center for Hematopoietic Diseases, Chonnam National University Hwasun Hospital, Gwangju, Jeollanam-Do Republic of Korea; 6 Division of Hematology, Department of Internal Medicine, Yonsei University College of Medicine, Seoul, Republic of Korea; 7 Division of Hematology-Oncology, Samsung Medical Center, Seoul, South Korea; 8 Department of Hematology, The Catholic University of Korea, Seoul, South Korea; 9 Department of Hematology-Oncology, School of Medicine, Kyungpook National University Hospital, Kyungpook National University, Daegu, South Korea; 10 Division of Hematology & Oncology, Department of Internal Medicine, Soonchunhyang University College of Medicine, Soonchunhyang University Hospital, Seoul, South Korea; 11 Department of Hematology-Oncology, Dong-A University College of Medicine, Busan, South Korea; 12 Division of Cancer Registration and Surveillance, National Cancer Control Institute, National Cancer Canter, Goyang, South Korea; 13 Department of Internal Medicine, Chonnam National University Hwasun Hospital, Chonnam National University, 322 Seoyang-Ro, Hwasun-Eup, Hwasun-Gun, Jeollanam-Do 58128 Republic of Korea

**Keywords:** Acute myeloid leukemia, Hematopoietic stem cell transplantation, Prognosis, Acute myeloid leukaemia, Acute myeloid leukaemia

## Abstract

This study aimed to validate the 2022 European LeukemiaNet (ELN) risk stratification for acute myeloid leukemia (AML). A total of 624 newly diagnosed AML patients from 1998 to 2014 were included in the analysis. Genetic profiling was conducted using targeted deep sequencing of 45 genes based on recurrent driver mutations. In total, 134 (21.5%) patients had their risk classification reassessed according to the 2022 ELN risk stratification. Among those initially classified as having a favorable risk in 2017 (*n* = 218), 31 and 3 patients were reclassified as having intermediate risk or adverse risk, respectively. Among the three subgroups, the 2022 ELN favorable-risk group showed significantly longer survival outcomes than the other groups. Within the 2017 ELN intermediate-risk group (*n* = 298), 21 and 46 patients were reclassified as having favorable risk or adverse risk, respectively, and each group showed significant stratifications in survival outcomes. Some patients initially classified as having adverse risk in 2017 were reclassified into the intermediate-risk group (33 of 108 patients), but no prognostic improvements were observed in this group. A multivariable analysis identified the 2022 ELN risk stratification, age, and receiving allogeneic hematopoietic cell transplantation as significant prognostic factors for survival. The 2022 ELN risk stratification enables more precise decisions for proceeding with allogeneic hematopoietic cell transplantation for AML patients.

## Introduction

Acute myeloid leukemia (AML) is a devastating and heterogeneous disease but is potentially curable^1^. Over the decades, substantial advances in the knowledge of AML and its genetic mutations have helped classify subgroups reflecting clinical characteristics and prognosis. Research on pathogenic mutations has also contributed to the recent development of novel therapeutic agents^2,3^. The European LeukemiaNet (ELN) has reported in the 2010 and 2017 editions the recommendations for diagnosing and managing AML^4,5^. These guidelines have been widely adopted by clinicians and investigators and have helped treat AML patients. Since 2017, there have been several advances in AML, including the interpretation of genetic abnormalities, the progress of measurable residual disease (MRD) techniques and their application to therapeutic responses and disease risk, and the development of novel therapeutic agents^2^. These advances have necessitated a new disease classification and updated the practice guidelines found in the 2022 ELN AML recommendations for diagnosing and managing AML in adults^2^. Compared with the previous recommendations, the new recommendations have several remarkable changes, especially in the risk stratifications of AML. First, the FMS-like tyrosine kinase 3-internal tandem duplication (*FLT3*-ITD) allelic ratio is excluded from the risk classification due to methodological issues in standardizing its analysis. Also, *FLT3-*ITD without a nucleophosmin 1 (*NPM1*) mutation is no longer classified as an adverse risk because of the disease-modifying effect of *FLT3* inhibitors^6–8^. Next, based on recent studies^9–11^, in-frame mutations affecting the basic leucine zipper region (bZIP) of CCAAT enhancer binding protein alpha (*CEBPα*) replaced the previous classification in which biallelic mutations of *CEBPα* were considered a favorable risk group. Another important change is the inclusion of myelodysplasia-related gene mutations other than *ASXL1* and *RUNX1*, and patients with these gene mutations (*BCOR*, *EZH2*, *SF3B1*, *SRSF2*, *STAG2*, *U2AF1*, and/or *ZRSR2*) are now categorized in the adverse risk group. These mutations are typically associated with secondary AML but are also prevalent in de novo AML and indicate adverse risk^12–15^. The presence of adverse-risk cytogenetic abnormalities in *NPM1*-mutated AML is classified as being an adverse risk^16,17^, and additional disease-defining recurring cytogenetic abnormalities, including t(3q26.2;v) and t(8;16)(p11.2;p13.3) were included in the adverse risk group^18^. Finally, hyperdiploid karyotypes with multiple trisomies (or polysomies) are no longer considered an adverse risk^19^.

However, there is insufficient research to determine whether these new guidelines are more practical than the 2017 ELN AML recommendations. This study aimed to verify the differences between the previous and new editions of the risk stratification of AML and to identify whether the 2022 ELN risk stratification is well reflected in the real world.

## Materials and methods

### Patient selection and study design

**Figure 1:**
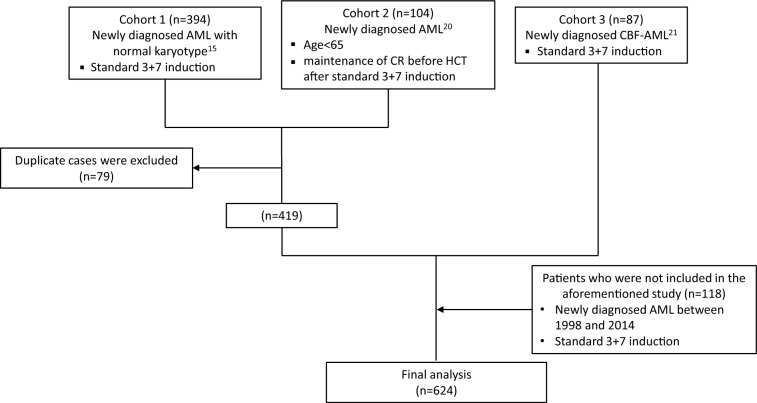
Flow chart of the included patients.

Patients who were newly diagnosed with AML, excluding those with acute promyelocytic leukemia (APL), from 1998 to 2014 were enrolled in this study. The current study cohort included a total of 624 patients and consisted of cohorts from our previous studies on core binding factor AML (*n* = 87), post-transplant measurable residual disease (MRD) monitoring (*n* = 104), and AML with secondary type mutations (*n* = 394)^15,20,21^. Among them, 79 duplicate cases were counted only once in the final analysis. Furthermore, an additional 118 AML patients from our institution, not included in any of the previous studies, were included in this study. The flow chart of the included patients is described in Fig. 1. All patients were older than 18 years old. All patients received intensive induction chemotherapy using a standard ‘7 + 3’ protocol (a three-day course of anthracyclines with a seven-day course of cytosine arabinoside [Ara-C] or N^4^-behenoyl-1-b-d-arabinofuranosylcytosine [BHAC]), and patients who achieved complete remission (CR) received consolidation chemotherapy. Since molecular genetic factors were not considered as they were not recommended at that time, patients who had favorable risk cytogenetics such as t(8;21)(q22;q22.1), inv(16)(p13.1;q22), or t(16;16)(p13.1;q22) did not proceed with allogeneic hematopoietic cell transplantation (HCT). The other patients were all treated with the intent to proceed with allogeneic HCT when matched donors were found.

The protocol was approved by the Institutional Review Board of Chonnam National University Hwasun Hospital (CNUHH-2014-083). All analyses were performed using medical records or cryopreserved samples, and written informed consent was obtained from all patients when they underwent bone marrow sampling for diagnosis. This research was performed in accordance with the Declaration of Helsinki.

### Analyses of genetic mutations

Genomic DNA was extracted using QIAamp DNA blood mini kits (Qiagen, Valencia, CA, USA) from cryopreserved bone marrow or peripheral blood samples obtained at diagnosis. Genetic profiling was performed for the targeted deep sequencing of 45 genes based on recurrent driver mutations identified in previous studies^12,22^. Regarding *CEBPα* mutations, in-frame mutations affecting the bZIP region of *CEBPα* were considered as a favorable risk group. Among the myelodysplasia-related mutations, *ASXL1* and *RUNX1* mutations were classified as adverse risks in both the ELN 2017 and ELN 2022 classifications. Agilent custom probes were designed to cover the whole exome regions of the targeted genes, and the genes were sequenced using an Illumina HiSeq 2000 sequencer. A variant allele frequency of 2% or more was defined as the cut-off for mutation positivity. All sequencing data have been deposited to the European Nucleotide Archive (Accession number: PRJEB49203)^15,20,21^.

### Statistical analyses

Categorical variables are presented as the number and percentage, and continuous variables are presented as the median and range. Discrete and continuous variables were evaluated using Fisher’s exact test and the Mann–Whitney U-test. Median follow-up was calculated using the reverse Kaplan–Meier method. Overall survival (OS) was measured from the initial diagnosis to death from any cause or the last follow-up. Event-free survival (EFS) was measured from the initial diagnosis to hematologic relapse or death from any cause. Relapse-free survival (RFS) was measured from CR achievement to hematologic relapse or death from any cause. OS, EFS, and RFS were estimated by the Kaplan–Meier method and compared using the log-rank test when comparing survival differences between two groups and the pairwise log-rank test when comparing survival differences between three groups. The Cox proportional hazards model using the Enter method was performed to analyze the hazard ratios (HRs) and 95% confidence intervals (CIs). Univariable analyses were performed with patient characteristics and clinical parameters, and all variables with a *p-*value less than 0.05 in the univariable analyses were included in the multivariable analyses. The collinearity diagnostics analysis and proportional hazards assumption test results using STATA/SE16 revealed that there were no significant interactions between the variables included in the multivariable Cox regression model, and the proportional hazards assumption was satisfied (Supplementary Tables 1, 2). Because of the confounding effect of allogeneic HCT on the survival of patients survival, an analysis of which patients were censored at the time of allogeneic HCT was conducted and presented in the Supplement material. To analyze the efficacy of allogeneic HCT, Cox proportional hazard regression with time-dependent covariates and a Mantel-Byar test were conducted using EZR software^23^.

To compare the survival predictions of each risk stratification group, C-statistics and net reclassification index (NRI) statistics were used. C-statistics and NRI statistics were performed using R software, version 4.2.2 (The R foundation for statistical computing, Vienna, Austria. https://www.R-project.org) and the ‘compareC’^24^ and ‘nricens’ packages^25^. A *p*-value of less than 0.05 was considered statistically significant. All statistical analyses without specific mentions were performed using SPSS (ver. 27; SPSS Inc., Chicago, IL, USA).

## Results

### Patient characteristics

A total of 624 patients were included in this study. According to the 2022 ELN risk stratification, patients were classified into favorable (*n* = 205, 32.9%), intermediate (*n* = 295, 47.3%), or adverse risk groups (*n* = 124, 19.9%). The characteristics of patients are summarized in Table 1. The median age of all patients was 51 years old (range, 18–84 years). There were no significant differences among the three groups in age, disease type, and the number of patients who received allogeneic HCT. After the induction of chemotherapy, 523 patients (83.8%) achieved CR. The CR rate of the favorable risk group was the best (92.2%), and the CR rate of the intermediate risk group (85.4%) was better than that of the adverse risk group (66.1%). Among the patients who achieved CR, 235 (44.9%) proceeded with allogeneic HCT. The proportions of allogeneic HCT among the three groups were not significantly different.

**Table 1:** Characteristics of patients.

2022 ELN	All patients n = 624		Favorable n = 205		Intermediate n = 295		Adverse n = 124		
	N	%	N (%)	%	n	%	n	%	*p*-value
Age, year									
Median (range)	51	(18–84)	47.5	(18–83)	50	(18–83)	57	(18–84)	
≥ 65 years	89	14.3%	25	12.2%	38	12.9%	26	21.0%	0.056
Sex									
Male	324	51.9%	102	49.8%	137	46.4%	85	68.5%	< 0.001
Disease type									
De novo AML	577	92.5%	194	94.6%	275	93.2%	108	87.1%	0.054
Secondary AML	34	5.4%	6	2.9%	15	5.1%	13	10.5%	
Treatment-related AML	13	2.1%	5	2.4%	5	1.7%	3	2.4%	
Genetic abnormalities									
t (8;21)	62	9.9%	62	30.2%	0	0.0%	0	0.0%	
inv(16) or t(16;16)	27	4.3%	27	13.2%	0	0.0%	0	0.0%	
t(9;11)	0	0.0%	0	0.0%	0	0.0%	0	0.0%	
t(6;9)	1	0.0%	0	0.0%	0	0.0%	1	0.8%	
t(v;11q23.3)	0	0.0%	0	0.0%	0	0.0%	0	0.0%	
t(9;22)	5	0.1%	0	0.0%	0	0.0%	5	4.0%	
t(8;16)	0	0.0%	0	0.0%	0	0.0%	0	0.0%	
inv(3) or t(3;3)	0	0.0%	0	0.0%	0	0.0%	0	0.0%	
t(3q26.2;v)	0	0.0%	0	0.0%	0	0.0%	0	0.0%	
− 5, del(5q); -7;-17/abn(17p)	7	1.1%	0	0.0%	0	0.0%	7	5.6%	
Complex karyotype, monosomal karyotype	17	2.7%	0	0.0%	0	0.0%	17	13.7%	
Mutated *NPM1* without *FLT3*-ITD	81	13.0%	81	39.5%	0	0.0%	0	0.0%	
Mutated *NPM1* with *FLT3*-ITD	67	10.7%	0	0.0%	61	20.7%	6	4.8%	
Wild-type *NPM1* with *FLT3*-ITD	48	7.7%	6	2.9%	48	16.3%	10	8.1%	
bZIP in-frame mutated *CEBPα*	37	5.9%	36	17.6%	0	0.0%	1	0.8%	
Mutated *ASXL1*, *BCOR*, *EZH2*, *RUNX1*, *SF3B1*, *SRSF2*, *STAG2*, *U2AF1*, *ZRSR2*	94	15.1%	16	6.8%	0	0.0%	78	62.9%	
Mutated *TP53*	19	3.0%	2	0.9%	0	0.0%	17	13.7%	
2017 ELN									
Favorable	218	34.9%	184	89.8%	31	10.5%	3	2.4%	< 0.001
Intermediate	298	47.8%	21	10.2%	231	78.3%	46	37.1%	
Adverse	108	17.3%	0	0.00%	33	11.2%	75	60.5%	
CR-achieved									
Yes	523	83.8%	189	92.2%	252	85.4%	82	66.1%	< 0.001
Allogeneic HCT (n = 523)									
Yes	235	44.9%	81	42.9%	112	44.4%	42	51.2%	0.592

*ELN* European LeukemiaNet, *AML* acute myeloid leukemia, *CR* complete remission, *HCT* hematopoietic stem cell transplantation.

**Figure 2:**
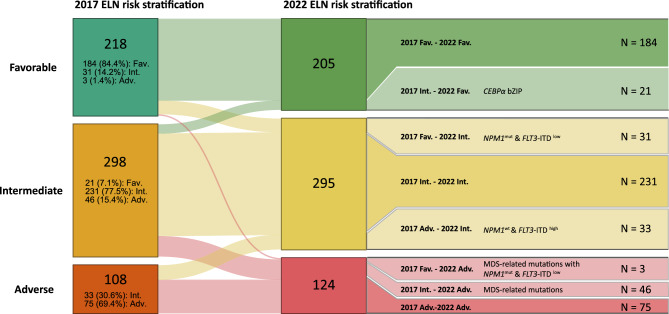
Risk group changes according to the 2017 and 2022 ELN risk stratifications. Risk group changes are presented using a Sankey plot. Presented genetic mutations are responsible for the risk group changes for each subgroup. *ELN* European LeukemiaNet, *Fav.* Favorable risk group, *int.* intermedaite-risk group, *adv.* adverse risk group, *CEBPα bZIP* basic leucine zipper region of CCAAT enhancer binding protein alpha, *NPM1* nucleophosmin 1, *FLT3-ITD* fms-like kinase 3-internal tandem duplication, *MDS* myelodysplastic syndrome.

According to the updated 2022 ELN risk stratification, 134 patients (21.5%) were reclassified to other risk groups (Fig. 2). In total, 218 patients who belonged to the 2017 ELN favorable risk group were reclassified into favorable (*n* = 184), intermediate (*n* = 31), and adverse risk (*n* = 3) groups according to the 2022 ELN recommendations. Of the patients previously in the intermediate risk group (*n* = 298), 21 were reclassified into the favorable risk group and 46 into the adverse risk group. Additionally, of the patients in the adverse risk group (*n* = 108), 33 patients were reclassified into the intermediate risk group. The numbers of patients with each genetic abnormality according to the 2022 ELN risk classification are presented in Supplementary Table 3.

### Survival outcomes of each group according to the 2017 and 2022 ELN risk stratification

The median follow-up duration was 84.4 months among the survivors. The five-year OS was 39.4% (95% CI 35.4–43.4), and the five-year EFS was 35.5% (95% CI 31.6–39.4) in all patients (*n* = 624). The five-year RFS was 42.5% (95% CI 38.1–46.9) in the patients who achieved CR (*n* = 523). Survival analyses according to the 2017 and 2022 ELN risk stratifications are shown in Supplementary Fig. 1. Each group, according to the 2017 and 2022 ELN risk stratifications, showed significant prognostic differences in OS, EFS, and RFS. Regardless of the 2017 or 2022 risk stratifications, the favorable risk group had the best prognosis, followed by the intermediate, then the adverse risk group.

### Comparison between the 2017 and 2022 ELN risk stratifications

To investigate which risk classification is better at predicting survival outcomes and prognosis, the HRs for each risk group were compared by each risk model. The survival differences of the subgroups when the 2022 ELN risk stratification was applied to each 2017 ELN risk group were analyzed. Additionally, the two risk stratifications using C-statistics and the NRI statistics were compared.

The HRs of each group compared to the favorable risk group are presented in Table 2. Applying the 2017 ELN risk stratification, the intermediate risk group had higher HRs than the favorable risk group, and the adverse risk group had the highest HRs for OS, EFS, and RFS. The differences were statistically significant. However, applying the 2022 ELN risk stratification, each risk group exhibited more noticeable differences in HRs than the previous risk stratification (Table 2).

**Table 2:** Hazard ratios of risk groups of the 2017 and 2022 ELN risk stratifications.

Hazard ratio (95% CI)						
	2017 ELN (total n = 624)			2022 ELN (total n = 624)		
	OS	EFS	RFS (n = 523)	OS	EFS	RFS (n = 523)
Favorable	*Reference*			*Reference*		
Intermediate	1.981 (1.476–2.659)	1.893 (1.431–2.503)	2.000 (1.432–2.792)	2.340 (1.702–3.216)	2.308 (1.705–3.124)	2.506 (1.758–3.574)
Adverse	3.391 (2.367–4.857)	2.973 (2.097–4.215)	3.376 (2.128–5.356)	4.380 (3.068–6.254)	4.083 (2.891–5.768)	4.201 (2.679–6.589)
*P*-value (log-rank)	< 0.001	< 0.001	< 0.001	< 0.001	< 0.001	< 0.001

*ELN* European LeukemiaNet, *CI* confidence interval, *OS* overall survival, *EFS* event-free survival, *RFS* relapse-free survival.

Among the patients classified into the favorable risk group by the 2017 ELN AML recommendations (*n* = 218), 34 (15.6%) patients were reclassified into other groups by the new risk stratification. All these patients had mutated *NPM1* with a low allele ratio of *FLT3*-ITD, three patients also had one or more myelodysplasia-related gene mutations, and one patient also had a biallelic but non-bZIP *CEBPα* mutation. Of the 31 patients assorted into the 2022 ELN intermediate risk group, three patients had myelodysplasia-related gene mutations and were reclassified into the adverse risk group.

**Figure 3:**
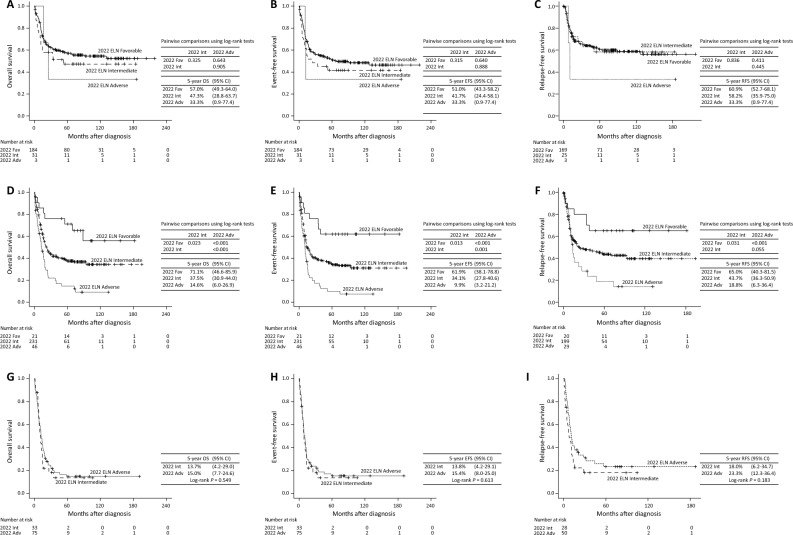
Prognostic differentiations according to the 2022 ELN risk stratification for each 2017 ELN risk group. The 2022 ELN risk stratification in the patients of the 2017 ELN favorable risk group; OS (**A**), EFS (**B**), and RFS (**C**). The 2022 ELN risk stratification in the patients of the 2017 ELN intermediate risk group; OS (**D**), EFS (**E**), and RFS (**F**). The 2022 ELN risk stratification in the patients of the 2017 ELN adverse risk group; OS (**G**), EFS (**H**), and RFS (**I**). *ELN* European LeukemiaNet, *CI* confidence interval, *Fav* favorable risk group, *Int* intermediate risk group, *Adv* adverse risk group, *OS* overall survival, *EFS* event-free survival, *RFS* Relapse-free survival.

When comparing the five-year OS according to the 2022 ELN risk stratification the favorable group had a five-year OS of 57.0% (95% CI 49.3–64.0), the intermediate group had a five-year OS of 47.3% (95% CI 28.6–63.7), and the adverse risk group had a five-year OS of 33.3% (95% CI 0.9–77.4) (*p* = 0.325 [favorable vs. intermediate], *p* = 0.643 [favorable vs. adverse], and *p* = 0.905 [intermediate vs. adverse]). When comparing the five-year EFS according to the 2022 ELN risk stratification the favorable group had a five-year EFS of 51.0% (95% CI 43.3–58.2), the intermediate group had a five-year EFS of 41.7% (95% CI 24.4–58.1), and the adverse risk group had a five-year EFS of 33.3% (95% CI 0.9–77.4) (*p* = 0.315 [favorable vs. intermediate], *p* = 0.640 [favorable vs. adverse], and *p* = 0.888 [intermediate vs. adverse]). When comparing the five-year RFS according to the 2022 ELN risk stratification the favorable group had a five-year RFS of 60.9% (95% CI 52.7–68.1), the intermediate group had a five-year RFS of 58.2% (95% CI 35.9–75.0), and the adverse risk group had a five-year RFS of 33.3% (95% CI 0.9–77.4) (*p* = 0.836 [favorable vs. intermediate], *p* = 0.411 [favorable vs. adverse], and *p* = 0.445 [intermediate vs. adverse]) (Fig. 3A–C).

When allogeneic HCT was censored, the OS, EFS, and RFS were well stratified in each subgroup according to the 2022 ELN recommendations. The favorable group had a five-year OS of 60.8% (95% CI 51.0–69.2), the intermediate group had a five-year OS of 15.7% (95% CI 1.3–45.4), and the adverse risk group had a five-year OS of 0.0% (*p* = 0.007 [favorable vs. intermediate], *p* = 0.203 [favorable vs. adverse], and *p* = 0.806 [intermediate vs. adverse]). The favorable group had a five-year EFS of 55.0% (95% CI 45.2–63.8), the intermediate group had a five-year EFS of 10.8% (95% CI 0.9–35.3), and the adverse risk group had a five-year EFS of 0.0% (*p* = 0.002 [favorable vs. intermediate], *p* = 0.135 [favorable vs. adverse], and *p* = 0.498 [intermediate vs. adverse]). The favorable group had a five-year RFS of 60.5% (95% CI 49.9–69.5), the intermediate group had a five-year RFS of 13.9% (95% CI 1.0–43.1), and the adverse risk group had a five-year RFS of 0.0% (*p* = 0.015 [favorable vs. intermediate], *p* = 0.015 [favorable vs. adverse], and *p* = 0.092 [intermediate vs. adverse]) (Supplementary Fig. 2A–C).

The number of patients classified to the 2017 ELN intermediate-risk group was 298. Of them, 21 (7.0%) and 46 (15.4%) were reclassified according to the 2022 ELN stratification to the favorable and adverse risk groups, respectively. All the patients reclassified into the favorable risk group had in-frame mutations in the bZIP region of *CEBPα*. Myelodysplasia-related gene mutations were the only reason patients were reclassified from the 2017 ELN intermediate risk group to the 2022 ELN adverse risk group. The OS, EFS, and RFS were well stratified according to the 2022 ELN stratification. The favorable group had a five-year OS of 71.1% (95% CI 46.6–85.9), the intermediate group had a five-year OS of 37.5% (95% CI 30.9–44.0), and the adverse risk group had a five-year OS of 14.6% (95% CI 6.0–26.9) (*p* = 0.023 [favorable vs. intermediate], *p* < 0.001 [favorable vs. adverse], and *p* < 0.001 [intermediate vs. adverse]). The favorable group had a five-year EFS of 61.9% (95% CI 38.1–78.8), the intermediate group had a five-year EFS of 34.1% (95% CI 27.8–40.6), and the adverse risk group had a five-year EFS of 9.9% (95% CI 3.2–21.2) (*p* = 0.013 [favorable vs. intermediate], *p* < 0.001 [favorable vs. adverse], and *p* = 0.001 [intermediate vs. adverse]). The favorable group had a five-year RFS of 65.0% (95% CI 40.3–81.5), the intermediate group had a five-year RFS of 43.7% (95% CI 36.3–50.9), and the adverse risk group had a five-year RFS of 18.8% (95% CI 6.3–36.4) (*p* = 0.031 [favorable vs. intermediate], *p* < 0.001 [favorable vs. adverse], and *p* = 0.055 [intermediate vs. adverse]) (Fig. 3D–F).

Among the patients in the 2017 ELN adverse risk group (n = 108), 33 patients (30.6%) were reclassified into the 2022 ELN intermediate risk group. All these patients had wild-type *NPM1* with a high allele ratio of *FLT3*-ITD mutations, which was one of the adverse risk factors in the previous recommendations. Patients in the 2017 ELN adverse risk group presented similarly poor survival rates even though they were reclassified by the new recommendations (Fig. 3G–I). When allogeneic HCT was censored, OS, EFS, and RFS showed similar results with uncensored analysis in intermediate and adverse risk group (Supplementary Fig. 3D–I).

Using C-statistics, Harrell’s C-index of the 2017 ELN risk stratification was 0.592 (95% CI 0.557–0.627), and that of the 2022 ELN risk stratification was 0.614 (0.579–0.649). The C-index of the new risk model was higher than the previous model, but it was not statistically significant (*P* = 0.059). In 134 patients whose risk groups were reclassified according to the ELN 2022 classification, the C-index of the 2017 ELN risk stratification was 0.527 (95% CI 0.460–0.594), and that of the 2022 ELN risk stratification was 0.599 (95% CI 0.526–0.672) (*P* = 0.158). The NRI method was used to compare each risk model and is summarized in Supplementary Table 4. In all patients, the estimated NRI at 12 months was positive, but the range of 95% CI included 0. However, as time passed, estimated NRIs became higher, and the NRIs at five years were all positive and in meaningful ranges. Further, when the patients who maintained the same risk group were excluded, the estimated NRIs tended to be higher (Supplementary Table 4).

### Efficacy of allogeneic hematopoietic stem cell transplantation

To investigate the role of allogeneic HCT according to the risk stratifications in AML patients, only patients who achieved CR were analyzed. The 2022 ELN favorable risk group had no survival difference between the allogeneic HCT and the non-allogeneic HCT subgroups. However, in the other risk groups, the allogeneic HCT subgroup had improved survival compared with the non-allogeneic HCT group (Supplementary Fig. 3). The differences between characteristics of patients who achieved CR according to allogeneic HCT are described in Supplementary Table 5. In total, 20 patients who achieved CR were included in the 2017 ELN intermediate risk group and were reclassified into the 2022 ELN favorable risk group. There were no survival benefits in the allogeneic HCT subgroup. All patients who changed from the favorable risk group to other groups had an *NPM1* mutant and a low *FLT3*-ITD allelic ratio. In those patients who changed from the 2017 ELN favorable risk group to a 2022 ELN non-favorable risk group, the allogeneic HCT subgroup showed a remarkable improvement in survival compared with the non-allogeneic HCT subgroup (Supplementary Fig. 4).

### Univariable and multivariable analyses

Univariable and multivariable analyses were performed using the 2022 ELN risk stratification, age, sex, white blood cell (WBC) count at diagnosis, blast percentage of bone marrow biopsy at diagnosis, and allogeneic HCT. The 2022 ELN intermediate-risk group had higher HRs and significantly worse survival outcomes than the favorable risk group (OS, HR 1.836 [95% CI 1.422–2.370], *p* < 0.001; EFS, HR 1.726 [95% CI 1.355–2.200], *p* < 0.001; RFS, 1.736 [95% CI 1.327–2.272], *p* < 0.001). The 2022 ELN adverse risk group had higher HRs than the favorable group (OS, HR 3.109 [95% CI 2.333–4.143], *p* < 0.001; EFS, HR 2.765 [95% CI 2.094–3.650], *p* < 0.001; RFS, HR 2.474 [95% CI 1.777–3.444], *p* < 0.001). According to the multivariable analysis, the 2022 ELN risk stratification, age, and allogeneic HCT were independent prognostic factors of AML (Table 3).

**Table 3:** Univariate and multivariate analyses.

	OS (n = 624)		EFS (n = 624)		RFS (n = 523)	
	HR (95% CI)	*P*-value	HR(95% CI)	*P*-value	HR (95% CI)	*P*-value
Univariate						
2022 ELN						
Favorable	*Reference*		*Reference*		*Reference*	
Intermediate	1.836 (1.422–2.370)	< 0.001	1.726 (1.355–2.200)	< 0.001	1.736 (1.327–2.272)	< 0.001
Adverse	3.109 (2.333–4.143)	< 0.001	2.765 (2.094–3.650)	< 0.001	2.474 (1.777–3.444)	< 0.001
Age (as a decade)	1.298 (1.204–1.399)	< 0.001	1.296 (1.205–1.393)	< 0.001	1.222 (1.125–1.327)	< 0.001
Sex						
Male	1.035 (0.844–1.268)	0.741	1.064 (0.873–1.297)	0.536	1.017 (0.809–1.278)	0.887
WBC (as log scale)	1.034 (0.881–1.214)	0.684	1.081 (0.924–1.265)	0.331	1.073 (0.894–1.289)	0.449
BM Blast (continuous)	1.002 (0.961–1.045)	0.913	1.006 (0.966–1.047)	0.787	1.016 (0.969–1.066)	0.504
Multivariate						
2022 ELN						
Favorable	*Reference*		*Reference*		*Reference*	
Intermediate	2.307 (1.677–3.173)	< 0.001	2.270(1.677–3.073)	< 0.001	2.471 (1.733–3.524)	< 0.001
Adverse	3.912 (2.733–5.601)	< 0.001	3.622 (2.558–5.129)	< 0.001	3.899 (2.480–6.128)	< 0.001
Age (as a decade)	1.245 (1.130–1.370)	< 0.001	1.249 (1.138–1.370)	< 0.001	1.177 (1.051–1.318)	0.005

*OS* overall survival, *EFS* event-free survival, *RFS* relapse-free survival, *HR* hazard ratio, *CI* confidence interval, *ELN* European LeukemiaNet, *WBC* white blood cells, *BM* bone marrow, *HCT* hematopoietic stem cell transplantation.

## Discussion

This study analyzed the changes between the 2017 ELN and 2022 ELN recommendations and their impact on patients. The new classification tends to stratify survival differences more accurately. Furthermore, this study verified that the updated risk classifications are more helpful in classifying patients who need allogeneic HCT.

Among the patients initially in the favorable risk group according to the 2017 ELN risk classification, there was no statistically significant survival difference between the 2022 ELN favorable risk group and the 2022 ELN intermediate and adverse groups (Fig. 3A–C). This result might be due to the confounding effect of allogeneic HCT because patients with a favorable risk received allogeneic HCT irrespective of the molecular risk stratification. Since allogeneic HCT could abrogate the adverse outcomes of patients who are reclassified as having an intermediate or adverse risk when the ELN2022 criteria are applied. In this study, all patients who changed from the favorable risk group to other groups had an *NPM1* mutant and a low *FLT3*-ITD allelic ratio. Allogeneic HCT was not recommended for these patients according to the previous 2017 ELN recommendations. The allelic *FLT3*-ITD ratio is no longer considered in the risk stratification, and the 2022 ELN recommendations explain that methodologically standardizing the *FLT3*-ITD ratio measurement is difficult^2^. However, several studies have questioned whether patients with low *FLT3*-ITD allelic ratios and an *NPM1*mutant are at a favorable risk. Sakaguchi et al. demonstrated that *NPM1-*mutant AML with a low *FLT3*-ITD allelic ratio is not associated with favorable outcomes, and survival of patients with this mutation is improved by allogeneic HCT^26^. Another retrospective study by Oran et al. found that allogeneic HCT in first CR improves outcomes irrespective of the *FLT3*-ITD allelic ratio^27^. This current study showed that allogeneic HCT improved the survival outcomes of patients with this mutation type (Supplementary Fig. 3C and D). These results suggest that *NPM1*-mutant AML with low *FLT3*-ITD allelic ratios is not a favorable risk factor, and patients with this mutation type require more treatment modalities such as a combination of *FLT3* inhibitors in addition to conventional induction and consolidation chemotherapies proceeding allogeneic HCT.

A cohort study that included 2,948 children and young adults with newly diagnosed AML showed that patients with a single bZIP domain mutation of *CEBPα* had similar outcomes to those with biallelic *CEBPα* mutations^9^. Two other retrospective studies showed that *CEBPα* mutations in the bZIP domain, whether monoallelic or biallelic, were associated with a favorable prognosis^10,11^. In this current study, all patients who changed from the 2017 ELN intermediate risk group to the 2022 ELN favorable risk group had in-frame mutations of the bZIP region of *CEBPα*. These patients presented good survival outcomes appropriate for the favorable risk group. Allogeneic HCT is not recommended at CR in favorable risk cytogenetics because it is not superior to consolidation chemotherapy and the risk of treatment-related mortality (TRM)^28,29^. Therefore, these patients were changed to a group that only administers consolidation chemotherapy rather than allogeneic HCT.

The patients in the 2017 ELN adverse risk group presented similarly poor survival rates when they were classified according to the 2022 ELN risk classification (Fig. 3G–I). Every patient who was reclassified into the 2022 ELN intermediate-risk group had wild-type *NPM1* with high *FLT3*-ITD allelic ratios. Because *FLT3* inhibitor-based therapy has improved survival, the new risk stratification defines the patients with this type of mutation as having an intermediate risk^2,6,7^. However, since *FLT3* inhibitors were not applied to the patients of this study, it is presumed that their prognosis was similar to that of the adverse risk group. More studies on the effect of *FLT3* inhibitors in such cases are needed.

After the 2022 ELN risk classification was published, various validations and discussions were expressed regarding the changed risk classifications^30–35^. The most controversial point is about the prognostic impact of myelodysplasia-related mutations. Myelodysplasia-related gene mutations, which were previously reported by Lindsley et al., such as *SRSF2*, *SF3B1*, *U2AF1*, *ZRSR2*, *ASXL1*, *EZH2*, *BCOR*, and *STAG2*, are related to chemoresistance of secondary AML and independent markers for adverse outcomes in de novo AML^36^. After that, several studies have reported the adverse prognosis of myelodysplasia-related mutations in AML^15,37–39^. A previous study reported that myelodysplasia-related gene mutations are independent markers for adverse outcomes in de novo AML, and this poor prognosis can be overcome by allogeneic HCT^15^. The ELN 2022 recommendations suggest that myelodysplasia-related mutations could not offset the favorable impact of *NPM1*. In this study, all patients with myelodysplasia-related mutations and mutated NPM1 without *FLT3*-ITD were classified as having a favorable risk. Recently, there have been some reports that have reported that myelodysplasia-related mutations are not consistently associated with poor prognosis^30,31^. However, in this study, all patients reclassified from other groups into the adverse risk group had myelodysplasia-related gene mutations and poor survival. The prognostic impact of myelodysplasia-related mutations should be further investigated in future studies.

Another point of discussion is the minimal residual disease (MRD) adjusted dynamic risk assessment. Jentzsch et al. reported that a significant proportion of patients in previously favorable or intermediate risk groups at diagnosis were reclassified after the MRD assessment but before allogeneic HCT^31^. This study did not have sequential data on MRD after induction treatment and transplantation, and therefore, the MRD-adjusted risk assessment was not considered. However, MRD is a powerful prognostic factor, and the MRD-based risk classification should be further studied and considered in the treatment of AML.

Despite some aforementioned limitations, many validation cohorts have agreed that the 2022 ELN recommendations more accurately stratify survival between patients^33,34^. The present study revealed consistent results by comparing the C-index of each risk stratification, and the 2022 ELN risk stratification was higher than that of the 2017 ELN risk stratification, although the *p*-value was not significant. The estimated NRIs at any time were positive, and the NRIs increased over time. After five years, all estimated NRIs and 95% CIs were positive. These trends indicate, in particular, that the new risk model more accurately reflects long-term survival. Although improvements in overall prognostic risk stratification could not be demonstrated, the 2022 ELN recommendations had the advantage of being able to further subdivide the prognosis of each ELN-2017 risk group.

This study was performed retrospectively and consisted of several different cohorts analyzed in the previously published studies. The cohort was composed of patients who received intensive induction treatment. Therefore, this cohort has limitations in accurately reflecting AML in the following areas: sex ratio, distribution of age, the proportion of each risk group, and the proportion of tAML or sAML. Another limitation is that this study specifically targeted patients eligible for 3 + 7 induction chemotherapy, resulting in a cohort of relatively younger individuals compared with other studies. This may contribute to the lower proportions of sAML and tAML in the present study^40,41^. In addition to age, which has traditionally been accepted as an independent prognostic factor, the association between male sex and adverse genetic risk has been gaining attention recently^30^. Therefore, the different demographic characteristics from the real AML population in this study might have influenced the results. However, since this study contains real-world data from a large number of patients with long follow-up periods, it meaningfully demonstrates the changed proportions from the previous 2017 ELN risk groups and the prognosis in the changed risk groups. Moreover, because the molecular genetic profiling was not reflected in the decision of allogeneic HCT, the prognostic characteristics of the molecular risk groups proceeding with transplants are demonstrated more prominently. Among the 624 patients, 134 (21.5%) patients were reclassified into other risk groups compared with the previous risk stratification. In total, 55 (8.8%) patients changed from the 2017 ELN favorable risk group to other groups or from other groups to the 2022 ELN favorable risk group. Based on this result, about 9% of patients can have changed treatment strategies at the transplant decision stage.

In conclusion, the recently updated 2022 ELN risk classification is helpful in segregating risk groups and predicting the prognosis of patients with AML. By applying the new recommendations, clinicians are advised to decide on treatment plans and provide more precise treatment strategies to AML patients according to their genetic abnormalities, particularly at the step of allogeneic HCT. Further extensive research is needed to verify that this risk stratification can be applied to elderly patients receiving low-intensity or molecular target therapy.

### Supplementary Information

Supplementary Information.

## Data Availability

All sequencing data have been deposited to the European Nucleotide Archive (Accession number: PRJEB49203). The datasets are available from the corresponding authors on reasonable request.
