# Supplementary material for: Validation of the 2022 European LeukemiaNet risk stratification for acute myeloid leukemia
Source: Sci Rep. 2024 Apr 12;14:8517. doi: 10.1038/s41598-024-57295-5 (PMC11014905; doi:10.1038/s41598-024-57295-5)
Supplement: Supplementary file 1 — Supplementary Information. [file 41598_2024_57295_MOESM1_ESM.docx]

**Validation of the 2022 European LeukemiaNet risk stratification
for acute myeloid leukemia**

Ga-Young Song^1*^, Hyeon-Jong Kim^1*^, TaeHyung Kim^2, 3, 4*^, Seo-Yeon Ahn^1^, Sung-Hoon Jung^1^, Mihee Kim^1^, Deok-Hwan Yang^1^, Je-Jung Lee^1^, Mi Yeon Kim^5^, June-Won Cheong^6^, Chul Won Jung^7^, Jun Ho Jang^7^, Hee Je Kim^8^, Joon Ho Moon^9^, Sang Kyun Sohn^9^, Jong-Ho Won^10^, Seong Kyu Park^10^, Sung-Hyun Kim^11^, Chang Kyun Choi^12^, Hyeoung-Joon Kim^1, 5^, Jae-Sook Ahn^1, 5^**‡**, and Dennis Dong Hwan Kim^2^**‡**

^1^Department of Hematology-Oncology, Chonnam National University Hwasun Hospital, Chonnam National University Medical School, Jeollanam-do, Republic of Korea; ^2^Department of Medical Oncology and Hematology, Princess Margaret Cancer Centre, University of Toronto, Toronto, ON, Canada; ^3^Department of Computer Science, University of Toronto, Toronto, ON, Canada; ^4^The Donnelly Centre for Cellular and Biomolecular Research, University of Toronto, Toronto, ON, Canada; ^5^Genomic Research Center for Hematopoietic Diseases, Chonnam National University Hwasun Hospital, Jeollanam-do, Republic of Korea; ^6^Division of Hematology, Department of Internal Medicine, Yonsei University College of Medicine, Seoul 03722, Republic of Korea; ^7^Division of Hematology-Oncology, Samsung Medical Center; ^8^Department of Hematology, The Catholic University of Korea, Seoul; ^9^Department of Hematology-Oncology, Kyungpook National University Hospital, School of Medicine, Kyungpook National University, Daegu, Korea; ^10^Division of Hematology & Oncology, Department of Internal Medicine, Soonchunhyang University College of Medicine, Soonchunhyang University Hospital, Seoul, Korea; ^11^Department of Hematology-Oncology, Dong-A University College of Medicine, Busan, Korea; ^12^Division of Cancer Registration and Surveillance, National Cancer Control Institute, National Cancer Canter

*These three authors contributed equally to this work.

Running title: 2022 ELN classification in AML

**‡Correspondence:**

Jae-Sook Ahn, MD, PhD

Department of Internal Medicine, Chonnam National University Hwasun Hospital, Chonnam National University

322 Seoyang-ro, Hwasun-eup, Hwasun-gun, Jeollanam-do, Republic of Korea, 58128

Tel: +82-61-379-7635 Fax: 82-61-379-8097 E-mail: f0115@chonnam.ac.kr

Dennis Dong Hwan Kim

Princess Margaret Cancer Centre, University of Toronto,

610 University Ave, OPG Rm 6-222, Toronto ON, Canada, M5G 2M9

e-mail: dr.dennis.kim@uhn.ca

**Supplementary Table 1. Collinearity diagnostics results for multivariate cox proportional hazards model**

| variable | VIF |
| --- | --- |
| 2022 ELN | 1.06 |
| Age (as a decade) | 1.25 |
| Sex | 1.03 |
| WBC (as log scale) | 1.13 |
| BM Blast (continuous) | 1.12 |
|  |  |
| mean VIF | 1.12 |
| Abbreviation: VIF, variance inflation factor; ELN, European LeukemiaNet; WBC, white blood cell; BM, bone marrow | |

**Supplementary Table 2. Test of proportional hazard assumption**

|  | Χ^2^ | *p*-value |
| --- | --- | --- |
| 2022 ELN |  |  |
| Favorable | . | . |
| Intermediate | 0.29 | 0.589 |
| Adverse | 0.32 | 0.569 |
| Age (as a decade) | 0.01 | 0.915 |
|  |  |  |
| total | 1.55 | 0.670 |
| Abbreviation: ELN, European LeukemiaNet | | |

**Supplementary Table 3. The number of patients with each genetic abnormality**

| **2022 ELN** | **Risk classification** | **N (%)** | **CR** | **Allo-HCT** |
| --- | --- | --- | --- | --- |
| **Favorable** | t(8:21)^1)^ | 62 (9.9) | 59 | 29 |
| **(n = 205)** | inv(16) or t(16;16)^2)^ | 27(4.3) | 26 | 8 |
|  | Mutated *NPM1* without *FLT3*-ITD^3)^ | 80 (12.8) | 71 | 23 |
|  | bZIP in-frame mutated *CEBPα*^4)^ | 36 (5.8) | 33 | 21 |
| **Intermediate** | Mutated *NPM1* with *FLT3*-ITD^5)^ | 61 (9.8) | 52 | 20 |
| **(n = 295)** | Wild-type *NPM1* with *FLT3*-ITD^5)^ | 48 (7.7) | 41 | 19 |
|  | t(9;11) | 0 (0.0) | 0 | 0 |
|  | Cytogenetic and/or molecular abnormalities not classified as favorable or adverse | 186 (29.8) | 159 | 73 |
| **Adverse** | t(6;9)^5)^ | 1 (0.2) | 1 | 1 |
| **(n = 124)** | t(v;11q23.3) | 0 (0.0) | 0 | 0 |
|  | t(9;22)^5)^ | 5 (0.8) | 5 | 4 |
|  | t(8;16) | 0 (0.0) | 0 | 0 |
|  | inv(3) or t(3;3) | 0 (0.0) | 0 | 0 |
|  | t(3q26.2;v) | 0 (0.0) | 0 | 0 |
|  | -5, del(5q); -7;-17/abn(17p)^5)^ | 7 (1.1) | 4 | 4 |
|  | Complex karyotype, monosomal karyotype^6)^ | 17 (2.7) | 14 | 11 |
|  | Mutated *ASXL1*, *BCOR*, *EZH2*, *RUNX1*, *SF3B1*, *SRSF2*, *STAG2*, *U2AF1*, *ZRSR2*^7)^ | 77 (12.3) | 50 | 18 |
|  | Mutated *TP53*^8)^ | 17 (2.7) | 8 | 4 |
| **Total** |  | 624 | 523 | 235 |

Abbreviation: ELN, European LeukemiaNet; CR, complete remission; Allo-HCT, allogeneic hematopoietic stem cell transplantation; *NPM1*, nucleophosmin member 1; *FLT3-*ITD, fms-like kinase 3-internal tandem duplication; bZIP, basic leucine zipper region; *CEBPα*, CCAAT enhancer binding protein alpha; *ASXL1*, additional sex combs like-1; *BCOR*, B-cell lymphoma 6 corepressor; *EZH2*, enhancer of zeste homolog 2; *RUNX1*, runt-related transcription factor 1; *SF3B1*, splicing factor 3b subunit 1; *SRSF2*, serine/arginine-rich splicing factor 2; *STAG2*, stromal antigen 2; *U2AF1*, U2 small nuclear RNA auxiliary factor 1; *ZRSR2*, zinc finger CCCH-type, RNA binding motif and serine/arginine rich 2; *TP53*, tumor protein p53

^1)^Mutated *NPM1* without *FLT3*-ITD (n=1), Wild-type *NPM1* with *FLT3*-ITD (n=2), Myelodysplasia-type mutation (n=3)

^2)^Wild-type *NPM1* with *FLT3*-ITD (n=2), Myelodysplasia-type mutation (n=1)

^3)^Myelodysplasia-type mutation (n=10), *TP53* (n=2)

^4)^Wild-type *NPM1* with *FLT3*-ITD (n=2), Myelodysplasia-type mutation (n=2)

^5)^No other genetic abnormality

^6)^Wild-type *NPM1* with *FLT3*-ITD (n=1)

^7)^Mutated *NPM1* with *FLT3*-ITD (n=6), Wild type *NPM1* with *FLT3*-ITD (n=9), bZIP in-frame *CEBPα* (n=1)

^8)^Myelodysplasia-type mutation (n=1)

**Supplementary Table 4. Net reclassification index (NRI)**

| NRI (95% CI) | | Overall survival (OS) | | Event-free survival (EFS) | | Relapse-free survival (RFS) | |
| --- | --- | --- | --- | --- | --- | --- | --- |
| All patients (Total n = 624, CR-achieved n = 523) | | | | | | | |
| At 12 months | | 173 cases, 214 controls | | 206 cases, 180 controls | | 124 cases, 159 controls | |
|  | NRI | 0.03059 | (-0.06775 to 0.13783) | 0.06273 | (-0.03005 to 0.15034) | 0.04467 | (-0.07083 to 0.15569) |
|  | NRI+ | 0.06083 | (-0.01491 to 0.13240) | 0.07603 | (0.00771 to 0.14046) | 0.04501 | (-0.03887 to 0.12697) |
|  | NRI- | -0.03024 | (-0.08276 to 0.01944) | -0.01329 | (-0.07126 to 0.03690) | -0.00034 | (-0.05594 to 0.05580) |
| At 24 months | | 235 cases, 133 controls | | 255 cases, 114 controls | | 158 cases, 108 controls | |
|  | NRI | 0.08956 | (-0.00976 to 0.18259) | 0.14397 | (0.05297 to 0.23553) | 0.14273 | (0.04067 to 0.24440) |
|  | NRI+ | 0.08065 | (0.02196 to 0.13901) | 0.09810 | (0.04548 to 0.15302) | 0.08608 | (0.02307 to 0.15079) |
|  | NRI- | 0.00891 | (-0.05435 to 0.07053) | 0.04587 | (-0.01672 to 0.10935) | 0.05665 | (-0.00341 to 0.12024) |
| At 60 months | | 259 cases, 71 controls | | 278 cases, 58 controls | | 177 cases, 57 controls | |
|  | NRI | 0.15432 | (0.05690 to 0.25237) | 0.20086 | (0.10913 to 0.29653) | 0.19109 | (0.09010 to 0.29932) |
|  | NRI+ | 0.09500 | (0.04433 to 0.14992) | 0.10186 | (0.05427 to 0.15214) | 0.09051 | (0.02789 to 0.15282) |
|  | NRI- | 0.05932 | (-0.00946 to 0.12404) | 0.09900 | (0.02961 to 0.16640) | 0.10059 | (0.03312 to 0.17003) |
| Reclassified patients (Total n = 134, CR-achieved n = 105) | | | | | | | |
| At 12 months | | 46 cases, 40 controls | | 54 cases, 32 controls | | 30 cases, 25 controls | |
|  | NRI | 0.07002 | (-0.35369 to 0.50299) | 0.22007 | (-0.20655 to 0.62699) | 0.18454 | (-0.35942 to 0.72225) |
|  | NRI+ | 0.23213 | (-0.08584 to 0.52246) | 0.29441 | (0.05137 to 0.54086) | 0.18647 | (-0.18777 to 0.50220) |
|  | NRI- | -0.16210 | (-0.42547 to 0.09673) | -0.07434 | (-0.35841 to 0.20795) | -0.00194 | (-0.31803 to 0.37736) |
| At 24 months | | 61 cases, 22 controls | | 65 cases, 18 controls | | 37 cases, 17 controls | |
|  | NRI | 0.37068 | (-0.08671 to 0.86675) | 0.67980 | (0.12603 to 1.19891) | 0.71314 | (0.14378 to 1.26393) |
|  | NRI+ | 0.31562 | (0.10520 to 0.52975) | 0.39138 | (0.18129 to 0.59487) | 0.36311 | (0.07259 to 0.60648) |
|  | NRI- | 0.05506 | (-0.31380 to 0.43414) | 0.28842 | (-0.16068 to 0.69922) | 0.35004 | (-0.05486 to 0.80855) |
| At 60 months | | 68 cases, 10 controls | | 73 cases, 7 controls | | 44 cases, 7 controls | |
|  | NRI | 0.81911 | (0.23002 to 1.44967) | 1.31485 | (0.67352 to 2.08999) | 1.25886 | (0.60371 to 2.08890) |
|  | NRI+ | 0.36699 | (0.17498 to 0.56305) | 0.39058 | (0.19651 to 0.58606) | 0.35268 | (0.11566 to 0.58267) |
|  | NRI- | 0.45212 | (-0.02273 to 1.03300) | 0.92427 | (0.34871 to 1.73599) | 0.90618 | (0.35414 to 1.67968) |

Abbreviation: CI, confidence interval; CR, complete remission; NRI, net reclassification index

**Supplementary Table 5. Characteristics of patients according to allogeneic HCT**

| **2022 ELN** | | **Favorable**  **n = 205 (CR, n=189)** | | | **Intermediate**  **n = 295 (CR, n=252)** | | | **Adverse**  **n = 124 (CR, n=82)** | | |
| --- | --- | --- | --- | --- | --- | --- | --- | --- | --- | --- |
|  |  | Allo-HCT(+)  (n=81) | Allo-HCT(-)  (n=108) | *p*-value | Allo-HCT(+)  (n=112) | Allo-HCT(-)  (n=140) | *p*-value | Allo-HCT(+)  (n=42) | Allo-HCT(-)  (n=40) | *p*-value |
| **Age, year** | Median (range) | 38 (15-62) | 53 (16-80) | <0.001 | 42 (15-65) | 55 (20-80) | <0.001 | 49 (18-67) | 61 (26-74) | <0.001 |
|  | <65 years | 81 (100.0) | 86 (79.6) | <0.001 | 111 (99.1) | 114 (81.4) | <0.001 | 40 (95.2) | 28 (70.0) | 0.003 |
|  | ≥65 years | 0 (0.0) | 22 (20.4) |  | 1 (0.9) | 26 (18.6) |  | 2 (4.8) | 12 (30.0) |  |
| **Sex** | Female | 40 (49.4) | 55 (50.9) | 0.884 | 60 (53.6) | 77 (55.0) | 0.899 | 17 (40.5) | 10 (25.0) | 0.163 |
|  | Male | 41 (50.6) | 53 (49.1) |  | 52 (46.4) | 63 (45.0) |  | 25 (59.5) | 30 (75.0) |  |
| **Disease type** | De novo AML | 79 (97.5) | 101 (93.5) | 0.229 | 107 (95.5) | 130 (92.9) | 0.688 | 67 (81.7) | 32 (80.0) | 0.015 |
|  | Secondary AML | 2 (2.5) | 3 (2.8) |  | 4 (3.6) | 7 (5.0) |  | 12 (14.6) | 7 (17.5) |  |
|  | Treatment-related AML | 0 (0.0) | 4 (3.7) |  | 1 (0.9) | 3 (2.1) |  | 3 (3.7) | 1 (1.2) |  |
| **2017 ELN** | Favorable | 70 (86.4) | 99 (91.7) | 0.339 | 13 (11.6) | 12 (8.6) | 0.704 | 1 (2.4) | 2 (5.0) | 0.009 |
|  | Intermediate | 11 (13.6) | 9 (8.3) |  | 86 (76.8) | 113 (80.7) |  | 9 (21.4) | 20 (50.0) |  |
|  | Adverse | 0 (0.0) | 0 (0.0) |  | 13 (11.6) | 15 (10.7) |  | 32 (76.2) | 18 (45.0) |  |

Abbreviation: ELN, European LeukemiaNet; AML, acute myeloid leukemia; CR, complete remission; HCT, hematopoietic stem cell transplantation

**
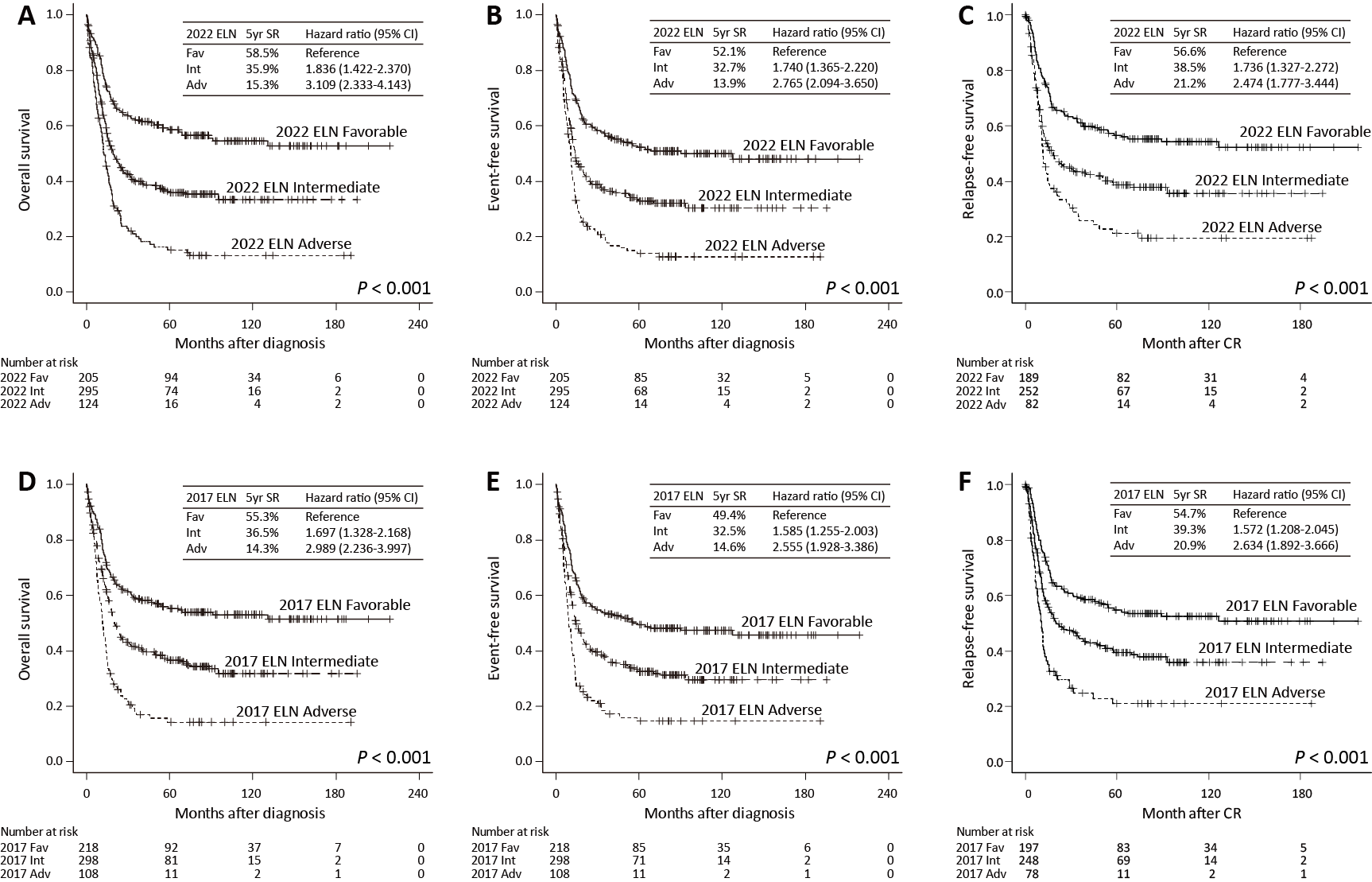
Supplementary Figure 1. Overall survival (OS), event-free survival (EFS), and relapse-free survival (RFS) according to the 2017 and 2022 ELN risk stratifications**

Abbreviation: ELN, European LeukemiaNet; 5yr SR, five-year survival rate; CI, confidence interval; Fav, favorable risk group; Int, intermediate risk group; Adv, adverse risk group

**Supplementary figure 2. Prognostic differentiation according to the 2022 ELN risk stratification in each 2017 ELN risk group when patients were censored at the time of allogeneic HCT.**

**
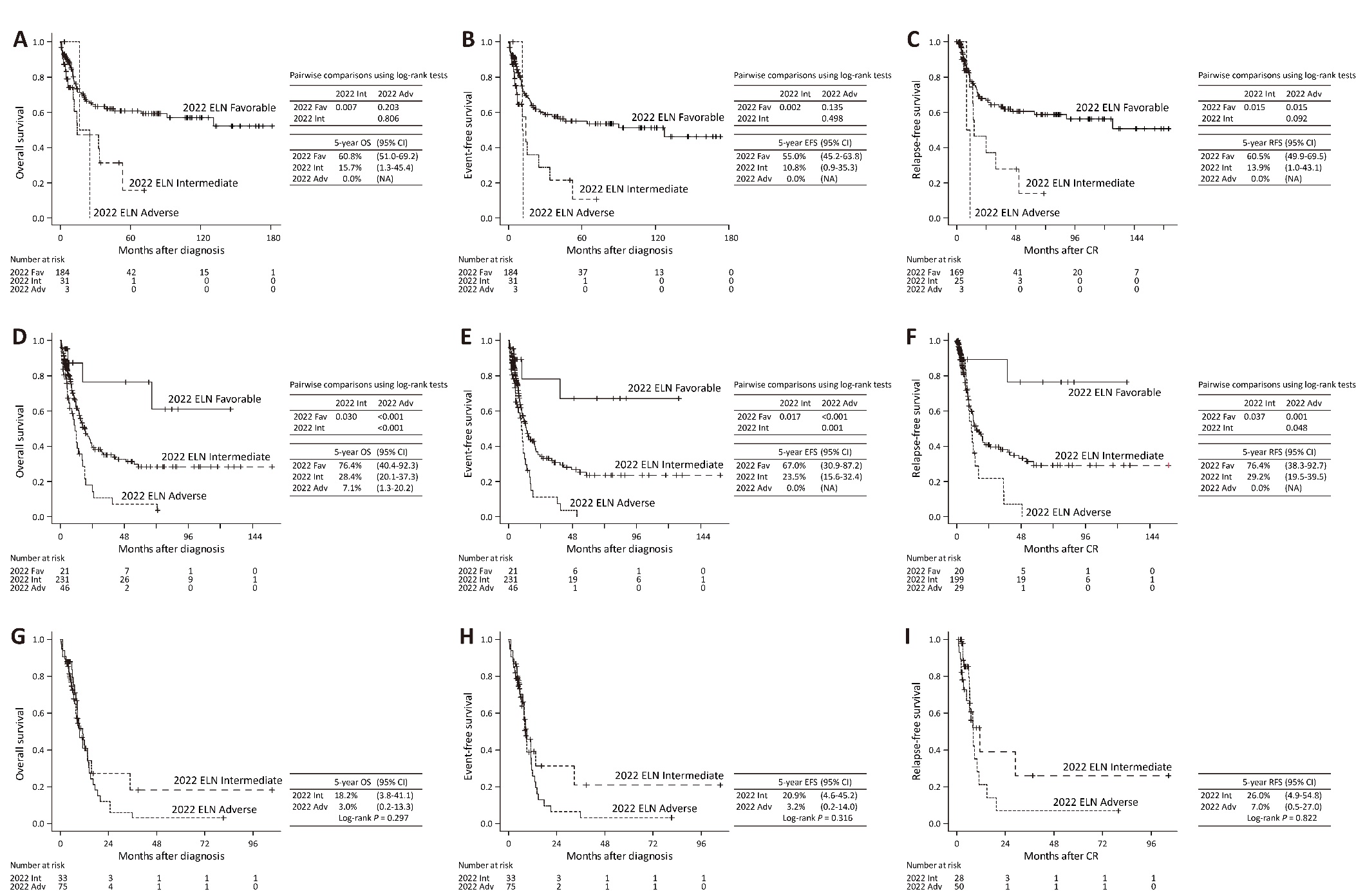
**


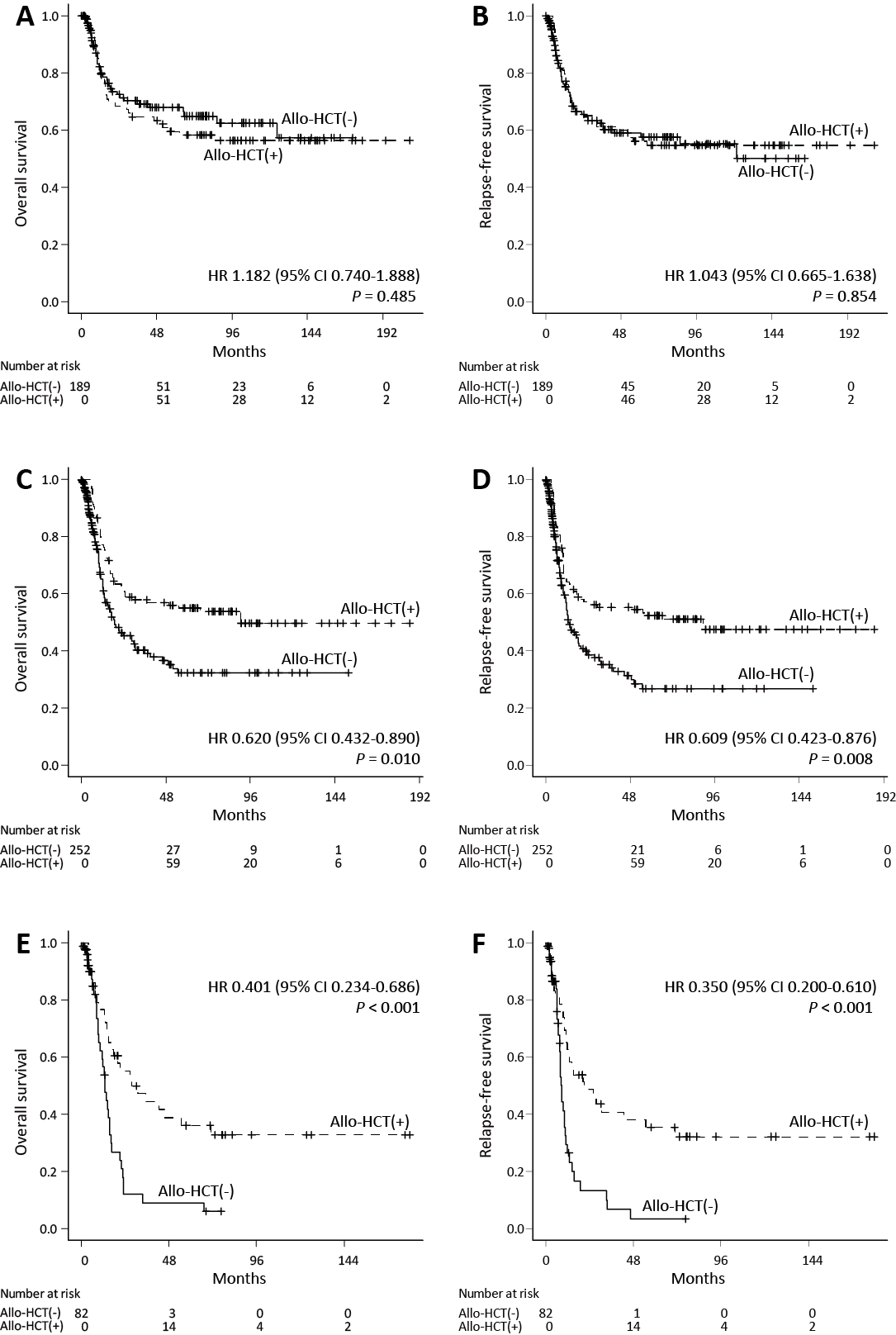
**Supplementary Figure 3. Efficacy analysis of allogeneic HCT in each risk group with Cox proportional hazard regression with time-dependent covariate and the Mantel-Byar test**

Survival difference between patients who received allogeneic HCT and did not in the 2022 ELN favorable risk group (A, B), in the 2022 ELN intermediate risk group (C, D), and in the 2022 ELN adverse risk group (E, F)

Abbreviation: Allo-HCT, allogeneic hematopoietic stem cell transplantation; HR, hazard ratio; CI, confidence interval


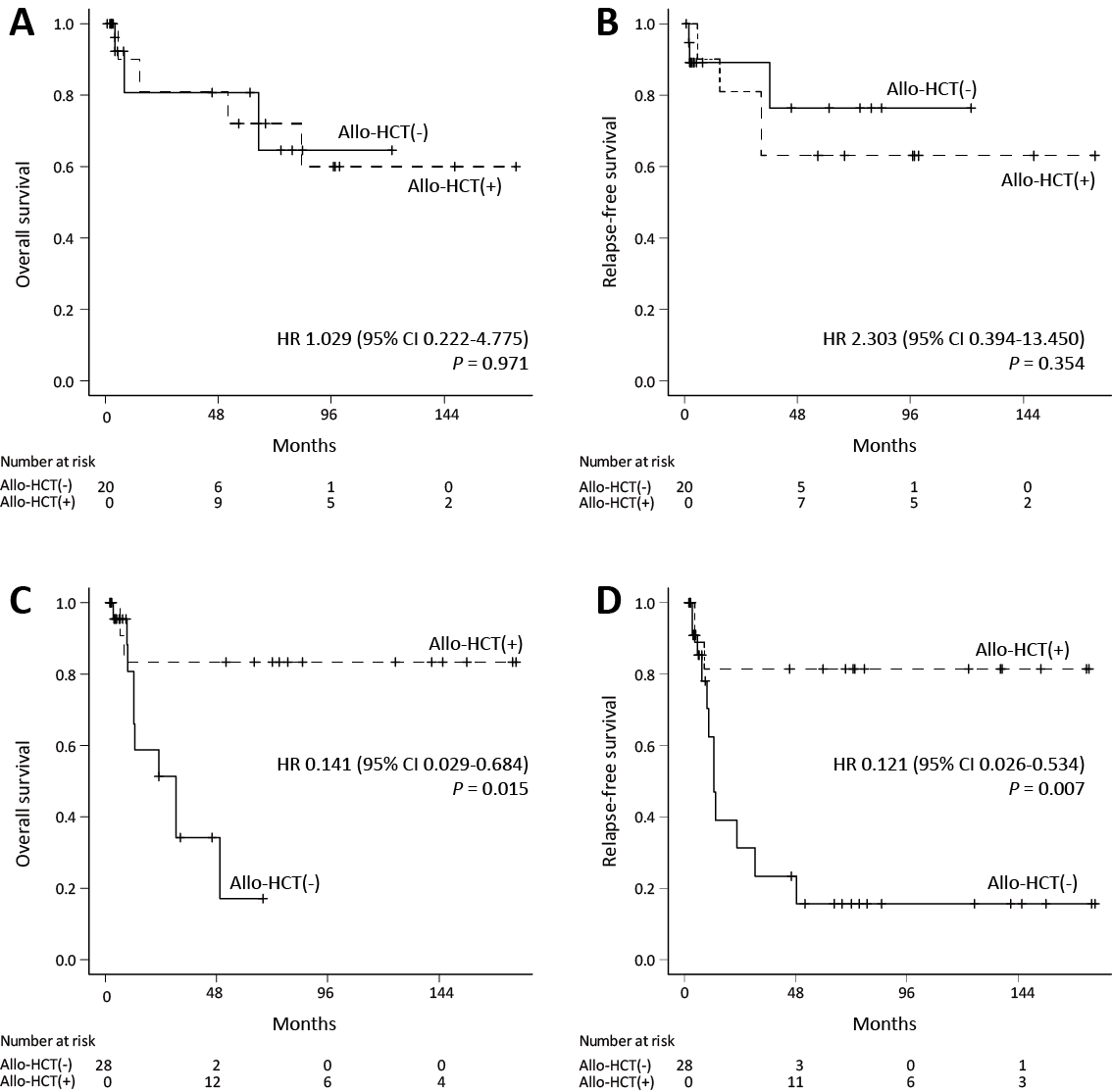
**Supplementary Figure 4. Efficacy of allogeneic HCT in the patients who were reclassified into the favorable risk group and the intermediate or adverse risk groups**

Patients who were reclassified into the 2022 ELN favorable risk group from the 2017 ELN non-favorable groups showed no survival difference between allogeneic HCT subgroup and non-allogeneic HCT subgroup (A, B). Patients who were reclassified into the 2022 ELN non-favorable risk groups from the 2017 ELN favorable risk group showed significant survival difference between allogeneic HCT subgroup and non-allogeneic HCT subgroup (C, D).

Abbreviation: Allo-HCT, allogeneic hematopoietic stem cell transplantation; HR, hazard ratio; CI, confidence interval

***Statistical analyses***

Categorical variables are presented as the number and percentage, and continuous variables are presented as the median and range. Discrete and continuous variables were evaluated using Fisher’s exact test and the Mann-Whitney U-test. Median follow-up was calculated using the reverse Kaplan-Meier method. Overall survival (OS) was measured from the initial diagnosis to death from any cause or the last follow-up. Event-free survival (EFS) was measured from the initial diagnosis to hematologic relapse or death from any cause. Relapse-free survival (RFS) was measured from CR achievement to hematologic relapse or death from any cause. OS, EFS, and RFS were estimated by the Kaplan-Meier method and compared using the log-rank test when comparing survival differences between two groups and the pairwise log-rank test when comparing survival differences between three groups. The Cox proportional hazards model using the Enter method was performed to analyze the hazard ratios (HRs) and 95% confidence intervals (CIs). Univariate analyses were performed with patient characteristics and clinical parameters, and all variables with a *p-*value less than 0.05 in the univariate analyses were included in the multivariate analyses. The collinearity diagnostics analysis and proportional hazards assumption test results using STATA/SE16 revealed that there were no significant interactions between the variables included in the multivariate Cox regression model, and the proportional hazards assumption was satisfied (Supplementary Table 1, 2). Because of the confounding effect of allogeneic HCT on the survival of patients survival, an analysis of which patients were censored at the time of allogeneic HCT was conducted and presented in the Supplement material. To analyze the efficacy of allogeneic HCT, Cox proportional hazard regression with time-dependent covariates and a Mantel-Byar test were conducted using EZR software (Kanda 2013). To compare the survival predictions of each risk stratification group, C-statistics and net reclassification index (NRI) statistics were used. C-statistics and NRI statistics were performed using R software, version 4.2.2 (The R foundation for statistical computing, Vienna, Austria. https://www.R-project.org) and the ‘compareC’ (Kang*, et al* 2015) and ‘nricens’ package(Pencina*, et al* 2011). A *p*-value of less than 0.05 was considered statistically significant. All statistical analyses without specific mentions were performed using SPSS (ver. 27; SPSS Inc., Chicago, IL, USA).

Kanda, Y. (2013) Investigation of the freely available easy-to-use software ‘EZR’ for medical statistics. *Bone Marrow Transplantation,* **48,** 452-458.

Kang, L., Chen, W., Petrick, N.A. & Gallas, B.D. (2015) Comparing two correlated C indices with right-censored survival outcome: a one-shot nonparametric approach. *Stat Med,* **34,** 685-703.

Pencina, M.J., D'Agostino, R.B., Sr. & Steyerberg, E.W. (2011) Extensions of net reclassification improvement calculations to measure usefulness of new biomarkers. *Stat Med,* **30,** 11-21.
